# Arsenic, Antimony, Chromium, and Thallium Speciation in Water and Sediment Samples with the LC-ICP-MS Technique

**DOI:** 10.1155/2015/171478

**Published:** 2015-03-22

**Authors:** Magdalena Jabłońska-Czapla

**Affiliations:** Institute of Environmental Engineering, Polish Academy of Sciences, M. Skłodowskiej-Curie 34 Street, 41-819 Zabrze, Poland

## Abstract

Chemical speciation is a very important subject in the environmental protection, toxicology, and chemical analytics due to the fact that toxicity, availability, and reactivity of trace elements depend on the chemical forms in which these elements occur. Research on low analyte levels, particularly in complex matrix samples, requires more and more advanced and sophisticated analytical methods and techniques. The latest trends in this field concern the so-called hyphenated techniques. Arsenic, antimony, chromium, and (underestimated) thallium attract the closest attention of toxicologists and analysts. The properties of those elements depend on the oxidation state in which they occur. The aim of the following paper is to answer the question why the speciation analytics is so important. The paper also provides numerous examples of the hyphenated technique usage (e.g., the LC-ICP-MS application in the speciation analysis of chromium, antimony, arsenic, or thallium in water and bottom sediment samples). An important issue addressed is the preparation of environmental samples for speciation analysis.

## 1. Introduction

The beginning of the 21st century is a time of great challenges in the analytical chemistry, which also includes the environmental analytics. Such a situation is mainly related to the new information on the toxicological properties of elements, their forms of occurrence, and the necessity to detect and determine lower and lower analytes levels, which are very often observed in the complex matrix samples. Speciation (a term borrowed from biology) describes the occurrence of various chemical and physical forms of a given element. The determination of such forms is known as speciation analytics [1]. Chemical speciation is an important subject in the environmental protection, toxicological and analytical research because toxicity, availability, and reactivity of trace elements depend on the chemical forms in which such elements occur. Two aspects can be differentiated within the speciation analytics framework, that is, determining man-made substances that are emitted into the environment by humans and analysing natural compounds formed as a result of biochemical transformations in the environment or living organisms. The first group is particularly interesting for the environmental analysis, whereas the other one concerns biochemists and ecotoxicologists. The fate and influence of trace elements are directly related to their chemical forms. They can occur as free ions, small organometallic formations, or bigger biomolecules included in the biological systems [1–5].

Due to the fact that metals and metalloids have a strong impact on the environment, the methods of their determination and speciation have received special attention in recent years. What is more, they have become one of the most important fields of application in the modern analytical chemistry. Arsenic, antimony, and thallium are examples of toxic elements.

Antimony is a very popular element in the environment, and its trivalent chemical species is about ten times more toxic as oxidized Sb(V) [6–10]. Another important and interesting metalloid is arsenic, whose inorganic species are much more toxic than organic ones [1, 11].

There are also elements that are very important for the health and life of living organisms.

Such an element is chromium, which reduced; inorganic form has a major role for the functioning of a living organism [12]. Unfortunately, hexavalent, oxidized chromium form is carcinogenic and mutagenic for humans. Similarly, thallium and its compounds are very toxic.

The element is also toxic in the dust form as it oxidizes in the contact with air. Food and respiratory thallium poisonings are possible. One of the characteristic poisoning symptoms is hair loss preceded by hair follicle atrophy. Other signs include digestion disorders, pain, neuropsychiatric complications, and cardiovascular system damage. In the past, thallium salts were often added to rodenticides [13]. The described elements have complex physical and chemical characteristics and are of great interest for both toxicologists and analytical chemists. Among them, arsenic and its compounds are the best known and described. Less information on antimony is available while thallium and it compounds are still the most mysterious and unfamiliar [14]. Unfortunately, the environmental pollution caused by human activity is still increasing, and hence the supply of metals and nonmetals is growing.

## 2. Speciation by Classical Methods or Rather by Using Hyphenated Techniques

The information obtained from toxicological tests and research into the influence of the specific chemical species on living organisms requires continuous lowering of the analyte detection limits to extremely low concentration levels. Such knowledge needs the development of the applied analytical methods. The progress enables the researchers to examine elements occurring at very low concentration levels and their chemical species, interactions, transformations, and functions in the biological systems. Such data is extremely important to understand the toxicology and metabolic routes of toxic elements, such as arsenic (As), antimony (Sb), chromium (Cr), or thallium (Tl).

Conventional methods are usually labor-intensive, time-consuming, and susceptible to interferences [15–20]. The most common tools for trace chemical speciation are the combination of separation techniques coupled with highly sensitive detector. In the early days, the separation consisted of a special off-line sample preparation followed by the detection step. The evolution and development of nonchromatographic methodologies based on chemical speciation are still growing because they can offer simple and inexpensive ways to made speciation, or, at least, for the determination of specific or toxic forms of trace elements [5]. The following analytical techniques are used in the thallium analytics: atomic absorption spectrometry, coulometry, spectrophotometry, ICP-MS, laser inducted fluorescence spectrometry, or differential pulse stripping voltamperometry [1].

The hyphenated techniques, in which separation method is coupled with multidimensional detectors, have become useful alternatives. The main advantages of those techniques consist in extremely low detection and quantification limits, insignificant interference influence, and high precision and repeatability of the determinations. Even though speciation analytics is relatively expensive, it plays an important role in the following fields: research into biochemical cycles of selected chemical compounds, determination of the toxicity and ecotoxicity of selected elements, quality control of food products and pharmaceuticals, control of technological processes, health risk assessment, and clinical analytics

In order to be able to continuously lower the detection and quantification limits, various separation and detection methods are combined. Such couplings are known as the hyphenated methods. Effective separation techniques for various chemical species and appropriate detectors are necessary to determine individual element forms. Most chromatographic methods, such as liquid chromatography (LC), are coupled with inductively coupled plasma-mass spectrometry (ICP-MS). ICP-MS offers many benefits, such as high element selectivity, broad linear range, and relatively low limit of detection (LOD). The basic separation mechanisms in the high-performance liquid chromatography (HPLC) that are applied in the environmental speciation analytics encompass the exclusion process, ion exchange, and chromatography in the reversed phase system. The use of the inductively coupled plasma collision cell-quadrupole mass spectrometry (ICP-CC-QMS), inductively coupled plasma dynamic reaction cell-quadrupole mass spectrometry (ICP-DRC-QMS) [2], or inductively coupled plasma-sector field mass spectrometry (ICP-SF-MS) [21–23] decreases the signal background (caused by molecular interferences) by separating the analyte signal from the signal of a given molecular ion.

Nonetheless, ICP-MS itself does not allow the researchers to obtain information on the chemical species of the examined element as full ionization of molecules in the plasma does not retain any molecular data. Liquid chromatography (LC), gas chromatography (GC), and capillary electrophoresis (CE) can be coupled with ICP-MS to determine various chemical species. Importantly, the coupling of CE with ICP-MS is not as the direct as the couplings with LC or GC. Coupling LC, GC, or CE (as separation methods) with ICP-MS opens up opportunities for the speciation analysis of elements in various samples. LC enables relatively simple coupling with the ICP spectrometer plasma torch without any major modifications in the standard system of the sample introduction in the ICP-MS spectrometer. LOD for liquid chromatography-inductively coupled plasma-mass spectrometry (LC-ICP-MS) may not be sufficient. Consequently, ultrasonic or pneumatic nebulizers [24, 25] can be used to improve LOD. One of the main limitations of LC-ICP-MS is the application of a suitable eluent. Only a mobile phase with the appropriate (limited) salt concentration and pH can be used. Importantly, it is advisable not to use organic solvents. Chromatographic techniques with the liquid mobile phase can be used to separate different chemical species, both in the off-line and on-line modes. When compared to the direct on-line separation of chemical species, the off-line separation has many disadvantages. The coupling of the isotopic analysis with the direct chromatographic separation can be performed with the multicollector-inductively coupled plasma-mass spectrometry (MC-ICP-MS). The advantages of the liquid chromatography-multicollector-inductively coupled plasma-mass spectrometry (LC-MC-ICP-MS) include sensitivity, selectivity, high ionization efficiency, and the ICP source resistance, which enables the coupling of chromatography and simultaneous monitoring of the relevant isotopes. At the same time, it provides the high precision of isotope correlations. Two separation methods are usually applied in LC-MC-ICP-MS, that is, ion-exchange columns or reversed phase [26].

## 3. Why Is Arsenic, Antimony, Chromium, and Thallium Speciation so Important?

Arsenic, antimony, chromium, and the underestimated thallium attract most interest of toxicologists and analysts. Their properties depend on the oxidation state in which they occur.

Antimony is common in the natural environment and comes both from natural processes and human activity. Over the years, the human activity brought about the significant increase in its concentration in the environment due to its applications in the car industry (i.a., as an additive in the car tyre vulcanization process). The geochemical behaviour of antimony is similar to that of arsenic and bismuth [6, 27, 28]. Its biological role is not fully recognized, but it is toxic at a low level (similarly to arsenic). Sb(III) is approximately 10 times more toxic than Sb(V). That is why there is such an interest in its speciation analysis [8, 9, 28]. Antimony and its salts mainly affect the central nervous system (CNS) and blood in the toxic way. They also cause conjunctivitis and skin inflammation and damage the heart muscle and liver. The antimony compounds demonstrate mutagenic and carcinogenic effects [27, 29].

Arsenic is a toxic metalloid that is common in various biological systems and the environment. The number of its speciation forms in the environment is still increasing due to the economic growth. As the industrial pollution has not been reduced in the recent decades, the arsenic emission from the industry, steelworks, animal waste, and the dust from fuel fossil combustion is currently rising. As arsenic is very mobile, it occurs in all the environment elements. The toxicity of arsenic itself and its compounds differs. However, its inorganic chemical species are about 100 times more toxic than the organic ones. The contact with arsenic can cause various health effects, such as dermatologic, inhalation, cardiologic, genetic, genotoxic, or mutagenic lesions [11]. It accumulates in the keratin-rich tissues, such as hair, skin, or nails. Arsenic and its inorganic forms can provoke cancers of the respiratory system or skin. They can also cause multiple organ cancer lesions. The dominant arsenic effects in humans are skin and mucous membrane lesions and nerve damage. Drinking water is one of the most important sources of the exposure to arsenic.

The most frequent poisonings are those caused by arsenic and its compounds. It has been used for approximately 1,000 years as the rodenticide because it is colourless and has neither taste nor smell. The toxic dose of arsenic is approximately 10–50 mg. It is lethal in the acute poisoning when the level is 70–200 mg or 1 mg/kg body weight. DMPS (2,3-Dimercapto-1-propanesulfonic acid) is validated in Germany as a medicine used in the acute or chronic mercury or lead poisoning (commercial names in Germany are Dimaval, oral capsules, and Heyl DMPS that is used for injections). In the USA, the DMPS application is considered experimental as the medicine has no approval of its effectiveness and safety granted by the U.S. Food and Drug Administration (FDA). Nonetheless, the reports presented in the literature prove its safety and effectiveness, when compared to other chelators. There is also a report that states that the oral application of DMPS is more effective than the injections. DMPS has been successfully used in the peripheral neuropathy caused by the arsenic poisoning [30].

Chromium is a classic example of an element whose two chemical species differ significantly in their chemical and toxicological properties. It is believed that the Cr(III) compounds have a positive influence on the functioning of living organisms. They are responsible for the appropriate glucose metabolism in mammals. They easily undergo complexation with various substances present in the environmental samples. On the other hand, the Cr(VI) compounds are extremely toxic. Their inhalation causes pneumonia and asthma, whereas their contact with skin provokes allergies and dermatoses [29]. The International Agency for Research on Cancer (IARC) classified the Cr(VI) compounds in the B-2 group, that is, substances carcinogenic and mutagenic for humans [31]. The Cr(VI) toxic effect results from its strong oxidising properties and also from the formation of free radicals in the reduction of Cr(VI) to Cr(III), which occurs in the cells. The Cr(VI) compounds are usually more easily soluble, mobile, and bioavailable, which maximizes their toxic effect. Even though the modern speciation analytics methods are developing fast, the standards and legal regulations still concern the total chromium and not its particular forms. Cr(VI) is 1,000 times more toxic than Cr(III), which is related to the fact that it easily penetrates the cell membrane (impermeable to the reduced chromium form). This ability results from the fact that the CrO_4_^2−^ ion is similar to the orthophosphoric and sulphate ions, which are transported in the appropriate ion channels into the interior of the cell. When the chromium ions are inside, they can react with the enzymes responsible for the metabolism of phosphate and sulphate ions. They can also react with DNA and RNA and disturb their normal functions. As a result, such reactions cause anomalies in the cell structure. The properties of chromium and its compounds and the methods used for their determination are described in detail in the study [32]. The literature examples of the Cr(III) and Cr(VI) ion determinations with the hyphenated methods are given in [33, 34].

Thallium was discovered by Sir William Crookes in 1861 and, independently, by Claude-Auguste Lamy in 1862. The element was introduced relatively quickly, that is, in 1880, as a medicine in the treatment of syphilis and mycosis. It was also used in depilation. Nonetheless, as thallium is highly toxic, its use was stopped at the beginning of the 20th century. Additionally, it has been abolished in pesticides in many countries in recent years as it was considered too toxic [35]. As the thallium application in various types of metal alloys has been increasing since the beginning of the digital revolution, it seems that the element has been accumulating in various elements of the environment. It is also used as a catalyst, in laser devices and in the production of optical fibres and high refractive index glass. The element occurs in two oxidation states, that is, +1 and +3. The Tl(I) compounds are colourless, and Tl(OH) is a strong and soluble base. The Tl(III) ions exist in the solution only when pH is close to 0. When it is higher, Tl(OH)_3_ precipitates [21]. The inorganic Tl(I) compounds are far more stable in a water solution with neutral pH than the Tl(III) compounds. On the other hand, the covalent organic thallium compounds are only stable for Tl (III). Each ionic form has different bioavailability and toxic properties. The Tl(III) cations are much more reactive and toxic than the Tl(I) ones. However, the number of Tl(III) cations is so low that Tl(I) is believed to be the most bioactive thallium form in the water environment, particularly for living organisms as it can replace the K+ ion. Thallium is highly toxic. Its average lethal dose for humans is 4–60 mg/kg. The Tl(III) toxicity is difficult to define because it is easily reduced in the biological systems [36]. The recent research has shown that Tl(III) can be even 50,000 times more toxic than Tl(I). Therefore, it is more toxic than Cd(II), Cu(II), Ni(II), or even Hg(II) [37, 38]. The Tl(I) salts are easily absorbed through skin and this is how they normally penetrate living organisms. Food is another source of thallium and its compounds. For this reason, food quality monitoring is very important at present. In clinical analyses, thallium is normally determined in urine, saliva, tissues, and blood. Balding preceded with the hair follicle darkening is a characteristic symptom of the thallium poisoning. Apart from these, digestion problems, psychological changes, and damage in the cardiovascular system occur. In the past, thallium salts were often used in rodenticides. Thallium is very common in the environment even though it usually occurs at very low concentration levels. The mean thallium concentration in the Earth crust is 0.3–0.5 mg/kg. Its content in soils is 0.02–2.8 mg/kg and depends on the geological bedrock composition and pollution. That is why the thallium contents vary in different countries (Austria, 0.076–0.911 mg/kg; China, 0.292–1.172 mg/kg; and Germany, in the vicinity of a lead and zinc mine, 8–27.8 mg/kg) [39].

## 4. Sample Preparation for Analyses

The analyte determination is one of the last stages of the analytical procedure that includes sampling, sample preservation, transport, storage, preparation for analyses, determination, and result processing. If the sample is collected, stored, or prepared for analyses in an inappropriate way, the most sophisticated analytical method and the most experienced analyst are not able to provide reliable results. The sample preparation stage is normally the most laborious part of the analysis. It is usually the most important source of errors. The sampling time should be as short as possible, which can be easily provided for water or bottom sediment samples. Factors that influence the analyte speciation in the real samples ought to be taken into account when storing the samples. For example, the storage of the samples for antimony determinations is very difficult, because Sb(III) easily transforms into Sb(V) in the oxidising environment [40]. To preserve the samples, the researchers often use chelating reagents, such as the ethylenediaminetetraacetic acid (EDTA). Studies on the stability of arsenic compounds in water samples chiefly concern the inorganic forms of this element, arsenite, and arsenate. There are many pieces of information about the redox stability of inorganic arsenic. The authors do not agree on the stability and permanence of arsenic forms in water, especially at different pH and in the presence of other substances [41]. Generally, in river water, As(V) is partially converted to As(III), but after 2 days, this is followed by gradual oxidation of As(III) into As(V) to reach an equilibrium. Storage at 5°C delays this oxidation by about 6 days [42].

In the case of thallium, diethylenetriaminepentaacetic acid (DTPA) (Merck) was used for stabilization of Tl(III) and sodium dodecyl sulfate (SDS) was used for extraction plant samples [43]. Other authors provide that river water samples, after sampling, were transported back to the laboratory and separation processes were finished within 8 hours of sample collection [39]. DTPS and HNO_3_ were used as extractants for the determination of Tl(I) and Tl(III) in the sediments of the Kłodnica River. This extractant was later used as an eluent during the chromatographic separation [44].

Trace elements can be present in the environmental samples at the ppb and lower concentration levels. It is often a great challenge for an analyst to extract the demanded analyte forms from the samples without changing their oxidation states. Additionally, sample storage is a very important issue in the speciation analysis. The environmental samples are normally frozen or stored in a refrigerator at 4°C and without the light access. Importantly, even such routinely used processes as dilution, changes in pH caused by the sample preservation, or pressure and temperature changes can bring about irreversible changes in the primary analyte form. There are particular difficulties when sampling takes place under conditions that differ significantly from those under which the sample is later analysed. The oxidation state change can occur in both directions due to the oxidation and reduction. For chromium, it is very unlikely (under normal conditions) to oxidise to Cr(VI), as Cr(III) oxidation to Cr(VI) takes place under drastic conditions (high temperature and oxygen presence or strong oxidation agent presence, such as Mn(IV) in a highly alkaline environment). It is very important to prevent the Cr(VI) reduction to Cr(III). For liquid samples (e.g., water samples), sampling, transport, and storage procedures should be as short as possible. Normally, the samples are frozen directly after sampling (transport). Such an action reduces the redox reaction kinetics [45].

Analysts often encounter problems related to the extraction of the suitable speciation analyte forms from the sample. It is particularly difficult when the analytes must be extracted from a complex matrix so that there are no changes in the oxidation state of a given chemical species. Usually, weak acids, buffers, or complexing reagents are used to extract inorganic or organic forms of low molecular weight. A proper extractant should not influence the analyte oxidation state and should be selected in such a way as to provide the highest extraction efficiency. The extraction efficiency test is performed through introducing an additive into the standard sample or extracting certified reference materials for soils or bottom sediments. It is assumed that the extraction procedure is correct, when the relative standard deviation (RSD) is ±5%. When the repeatability of results is poorer, there is no process control [46].

The use HPLC as time-resolved introduction techniques into the atomic spectrometer establishes some physicochemical requirements for the analytes. This usually makes a sample preparation procedure that includes the pretreatment of the sample with some type of reagent to condition the matrix or leach the species for the extractions step in which the species are completely isolated from the matrix necessary.

Most living organisms reduce the toxicity of arsenic and antimony by incorporating them into organometallic molecules through metabolic pathways [41]. Therefore, speciation methods have to be capable of extracting these compounds without structural modifications. More ubiquitous organoarsenic environmental molecules are monomethylarsonic acid (MMA), dimethylarsinic acid (DMA), arsenobetaine (AsB), and arsenocholine (AsCh) and extraction procedures can be developed to isolate them from matrices. Due to the stability of methylated arsenic species, they are leached together with total inorganic arsenic, using warm [47] or cold [48] concentrated HCl from sediments and biological tissues. Arsenic chemical species could be leaching using acidic solvents (pH = 2.3) for As(III) or basic leaching (pH = 11.9) for As(V), MMA, and DMA. Other weak leaching reagents such as acetate, citrate, and oxalate buffers selectively leach As(III) and phosphoric acid efficiently extracts total arsenic from soils [41]. Phosphate buffer (5 mM Na_2_HPO_4_/50 mM NaH_2_PO_4_ (pH = 6,0) and ultrasonic bath were used for extraction of inorganic arsenic species in sediment samples [49]. As most of the arsenic is usually associated with iron oxides, a selective extraction method using hydroxylammonium hydrochloride as extractant with special emphasis on the different arsenic chemical species (As(III), As(V), MMA, and DMA) in the extract has been performed [50].

## 5. Application of Chromium, Arsenic, Antimony, and Thallium Speciation Analyses in Water Samples

Generally, it is known that the contents of chromium, arsenic, antimony, and thallium are very low in the uncontaminated samples. It is necessary to use very sensitive methods, such as ICP-MS, to determine such low analyte contents [51]. Over the last two decades, there have been many studies concerning the determination methods and occurrence of Cr(III) and Cr(VI) in the natural environment. The chromium chemistry is very complex. The concentration of oxidising substances is another important factor that affects the chromium redox behaviour. Even though there are a few substances that are able to oxidise Cr(III), only a few of them have sufficiently high concentrations that enable the oxidation of Cr(III) to Cr(VI) in the environment. The situation is different when compared to the Cr(VI) reduction to Cr(III). In this case, the concentrations of the reducing substances are high enough and play the main part even though the reduction is less thermodynamically privileged. The precipitation and dissolution processes also influence the contents of the chromium chemical species [12].

The research into chromium speciation with hyphenated techniques is very popular. Bednar et al. [32] examined various water types (surface, ground, and tap water) with the anion-exchange AG-11 and AS-11 columns (Dionex) coupled with the ICP-MS detection. There was also the research into three water reservoirs (Pławniowice and Goczałkowice Reservoirs, and Rybnickie Lake) that differed in the anthroporessure type. The chromium content in the Pławniowice reservoir demonstrated variations in the chemical species concentrations, which depended on the sampling month. Cr(III) dominated in winter and spring months, whereas the Cr(VI) dominance was observed in the surface water in June (probably related to the oxygen content of 135%). The Cr(III) concentration in the bottom water was the lowest in July–October period. There was also a strong correlation between the Cr(VI) concentration and pH in the bottom water [33]. Cr(III) also dominated the chromium content in the Goczałkowice reservoir and Rybnickie Lake. The water research indicated the seasonal variations in the concentrations of the chromium chemical species. The high oxygen content and highly reducing conditions were also responsible for the lack of Cr(VI) in the porous water of the bottom sediments [34], which were collected from a river near a tannery.

It is necessary for the hyphenated methods used in the arsenic speciation analytics (at low concentration levels) to be both appropriately selective and sensitive. There are many studies in the literature on the instrumental methods used in the speciation of 5 arsenic chemical species. Most of them are based on the chromatographic separation techniques, such as HPLC [52].

The researchers determined arsenic chemical species in the Ohio River water samples with the ion-pairing reversed phase chromatography with inductively coupled plasma-mass spectrometry (IPRP-ICP-MS) with tetrabutylammonium hydroxide (TBAH) and C-18 column. The obtained LODs were at the ng/L level [53]. Bednar et al. [54] examined surface, ground, and even acidic waters from a coal mine with the hyphenated system of HPLC-ICP-MS. They used various columns and eluents.

In 1990s, there were many successful studies into the chromium [55] and arsenic [56, 57] chemical species and subsequently into the simultaneous determination of the chromium and arsenic speciation forms [58]. At the beginning, only water samples were examined and the obtained LODs were at the *μ*g/L level.

HPLC coupled with a sector mass analyser and ICP-MS was used for the arsenic speciation in the environmental samples collected from Moira Lake and the Moira River (Ontario, Canada) [59]. The researchers proved that the Moira River water was highly polluted with arsenic, particularly in summer. The total arsenic content in the river water exceeded 140 mg/L. On the other hand, the arsenic concentration in the Moira Lake water was 40–50 mg/L. The arsenic speciation proved that As(V) was dominant in the surface water. The content of the totally dissolved As(III) in water was approximately 2% of the total content. Such results are typical for waters with a high oxygen content and fast flow. Nonetheless, As(III) was dominant in the bottom water. The analysis suggests that three processes affect the distribution of As(III) and As(V) in the water column, that is, diffusion of As(V) from the interstitial waters to the upper layers; As(III) formation resulting from biological transformations; and dissolution of the suspension and atmospheric dusts that contain As(III).

As(V) was also the main arsenic chemical species in the Kamo and Ichonakawa Rivers (Japan) [60]. The study indicates similarities between the geochemical behaviour of arsenic and antimony under the oxygen conditions in the researched rivers.

The stability of the environmental and biological samples, and particularly waters, in the speciation analysis largely depends on the sample matrix. The published data demonstrates that storing water samples at 5°C delay the oxidation of As(III) to As(V), whereas acidification can affect the changes in the distribution of particular chemical species in the samples. The performed research clearly shows that approximately 10% of As(III) oxidizes to As(V) under the sample matrix effect after 24 hours of the surface water sample storage. After the 3-day storage, 10–90% of As(III) turns into As(V), which depended on the sample. At the same time, the research into the Cr(VI) content in the same samples reveals that its concentration did not change even after 15 days [61, 62].

The arsenic speciation in the surface and well waters showed that arsenites and arsenates were the main forms found in the samples. In the surface waters, both DMA(V) and AsB were also observed. The presence of the methyl derivatives is probably related to the occurrence of microorganisms. No methyl derivatives of arsenic were observed in the well and mineral waters [52].

There are also publications which discuss simultaneous determinations of the chemical species of various elements. For example, in the study [61], the authors researched arsenic speciation forms and Cr(VI) using HPLC-ICP-MS with the anion-exchange Hamilton PRP-X100 column. The application of HPLC-ICP-MS in the arsenic speciation for water samples was described in detail in the study [63].

In China, antimony speciation was performed with HPLC-ICP-MS to examine water from the biggest antimony mine in the world. The authors determined Sb(V) and Sb(III) in the samples. It turned out that the Sb(V) form was the dominant one. Only trace amounts of Sb(III) were found [64]. Asaoka et al. [60] obtained similar results. They examined arsenic and antimony chemical species in the waters and bottom sediments of the Kamo and Ichinokawa Rivers (Japan). They observed that Sb(V) was the dominant speciation form in each water sample. It was probably dissolved as Sb(OH)_6_^−^.

Analysts also investigated hot spring water sold as drinking water to determine the contents of the arsenic and antimony chemical species with HPLC-ICP-MS [65]. The researchers observed that only inorganic arsenic species, such as As(III) and As(V), were present in the analysed waters. No antimony chemical species were found.

In another study, tap water was examined and inorganic antimony chemical species were determined in the samples. Sb(V) was the dominant form, while the Sb(III) content was below LOD [66]. Similarly [67], when tap water was researched, it was observed that the mean Sb(V) concentration was 20 ng/L, whereas the Sb(III) and TSbCl_2_ contents were below the method LOD.

Other authors used the complexation reactions to form stable Sb(III) and Sb(V) complexes, which were afterwards separated in the HPLC-ICP-MS system with the PRP-X100 column. The obtained low LODs were 0.05 *μ*g/L for Sb(III) and 0.07 *μ*g/L for Sb(V) [68].

Apart from the antimony inorganic chemical species, trimethylstiboxide (TMSbO) was examined in the surface water [69]. TMSbO is stable in water and can be reduced to trimethylantimony (TMSb). It can be formed either in the bacteriological process (e.g., in the soil) or during trimethylantimony oxidation in the biomethylation process of the antimony compounds. Waters polluted due to the industrial activities and mining processes were investigated. The researchers found the contents of Sb(V) and Sb(III) at the levels of 90% and 10%, respectively. No TMSbO was found in the polluted water samples. Nevertheless, some chromatograms showed peaks of the unknown antimony species that originated from certain stable antimony complexes.

The research into the contents of the arsenic, antimony, and chromium chemical species is very popular. The same situation is observed for the analyses of thallium speciation forms in the environmental samples [70, 71]. The application of HPLC-ICP-MS enables determining thallium species in the samples of the sea [72] and surface [14, 44] waters. The flow injection analysis coupled with atomic absorption spectrometry (FIA-AAS) is another technique often applied in the thallium speciation [73]. However, the most popular, in the case of thallium, are speciation methods of combining extraction procedures with very sensitive detection techniques [74]. In this study, a simple and novel sequential mixed micelle cloud point extraction procedure for the separation of Tl species in environmental water samples for their determination by ICP-MS, without using any additional salts or chelating agents was used. The anionic mixed micelle comprising sodium dodecyl sulfate (SDS) and Triton X-114 is used for selective extraction of positive Tl(III), DTPA species into the surfactant-rich phase. To improve the preconcentration factor, ultrasound was used for back-extraction of Tl(III). Other authors studied thallium speciation in river waters, using Chelex-100 resin and atomic absorption spectroscopy technique [39].

## 6. Application of Chromium, Arsenic, Antimony, and Thallium Speciation Analyses in Bottom Sediment Samples

The high chromium content in bottom sediments is often caused by the close vicinity of tanneries, steelworks, or galvanic shops. The tanning industry is a typical source of Cr(III), including mainly sulphates [34]. Under the redox and slightly oxidising conditions, Cr(VI) is reduced to Cr(III) within the period that ranges between a few minutes and a few days. Cr(III) is the chromium chemical species that is most often adsorbed on bottom sediments. The Cr(VI) adsorption is significantly lower than that of Cr(III). It depends on pH and occurs more easily under acidic conditions.

Jabłońska et al. [33] investigated bottom sediments sampled from the Pławniowice and Goczałkowice Reservoirs and Rybnickie Lake. In the Pławniowice Reservoir [60], the bottom sediment analysis indicated high contents of the easily leached fractions (metals in the porous solution, carbonate, and ion-exchange fractions). The chromium speciation analysis of the Pławniowice bottom sediment revealed slight dominance of its reduced form (Cr(III), 56%; Cr(VI), 44%). In Rybnickie Lake, the high Cr(VI) content was observed in the bottom sediment, which was most probably related to the phytoplankton bloom. Phytoplankton is able to accumulate, i.a., chromium (particularly Cr(VI)), both inside the cell and on its surface (phytosorbent). The organic matter that lands on the lake bottom enriches the Rybnickie Lake bottom sediments with Cr(VI). The chromium speciation analysis in the easily leached fractions demonstrated significant dominance of the oxidised form, Cr(VI), whose percentage in the heated water discharge zone and dam zone was 75% and 62%, respectively.

In the study [69], the authors focused on the sample preparation methods. They particularly concentrated on the extraction of the solid samples (including bottom sediments) for the analysis with HPLC-ICP-MS. The harbour water and sediments (Baltimore, USA) had low concentrations of Cr(VI), which was reduced to Cr(III) under the conditions existing in the harbour. The application of the Brownlee C8 column in the HPLC-ICP-MS system helped to determine highly saline samples [75].

Inorganic arsenic compounds are the most toxic arsenic forms that occur naturally in the environment. The arsenate toxic effect results from the mechanism of oxidative phosphorylation uncoupling. The research into the contents of the arsenic chemical species in Lake Moira, which is one of the biggest lakes in Canada, indicated the complexity of the undergoing processes. The total arsenic concentration in the bottom sediments was determined after acid digestion. The result was many times higher than the background value. The arsenic extraction from the bottom sediments was performed with the mixture of the phosphoric acid (1 mol/L) and ascorbic acid (0.1 mol/L). The concentration of the arsenic species was determined in the HPLC-ICP-SF-MS system. It was observed that the As(III) concentration decreased with the increasing depth of the particular bottom sediment layers. The As(III)/As-complex ratio in the extracts also indicated the tendency to decrease with the increasing depth. The highest As(III)/As-complex ratio was obtained in the surface layer of the Lake Moira bottom sediments. The authors suggest that As(III) was released from the surface layer of the bottom sediments in the redox or decomposition process. Subsequently, it was moved into water through the bottom sediment/bottom water exchange. The research points to the complexity of the forming organic species of arsenic and the necessity to investigate fresh, not dried, and bottom sediments [59].

In another study, 0.3 M phosphoric acid was used as the extractant of the arsenic chemical species from the bottom sediment samples that were determined with the HPLC-ICP-MS system [76].

The research into the bottom sediments of the Godavari River Estuary (the third biggest river in India) shows that the increase in the salinity of the water column above the bottom sediments also affects the arsenic distribution and speciation in the sediments. The researchers determined the As(III) and As(V) with the spectrophotometric methods. They also used sequential extraction procedure proposed by the Community Bureau of Reference (BCR) [77].

The concentrations of arsenic and antimony in bottom sediments are often correlated. The research demonstrates that the Sb(V) content is 60–84% of the total antimony content. The authors point to the important adsorption influence on the arsenic and antimony concentrations in the bottom sediments. They also reveal that the distribution and migration of arsenic and antimony in the water bottom sediment system were similar under the oxygen conditions observed in the river. When taking into consideration the redox conditions in the river, it is not surprising that As(V) and Sb(V) forms dominated. The coefficients for arsenic and antimony in water and bottom sediments were similar (approximately 4.7 at Eh > 200 mV) [59].

The literature does not provide many reports on the thallium speciation in bottom sediments with HPLC-ICP-MS [44]. Most investigations concern bottom sediment fractionation [78] and extraction of particular chemical species [79, 80].  Table 1 presents the application of LC-ICP-MS techniques in chromium, arsenic, antimony, and thallium speciation in water and sediment samples.

## 7. Conclusions

Even though speciation analytics has been rapidly developing over the last 30 years, it is still a relatively new field of the analytical chemistry. Its further progress depends on many factors, such as the new sample preparation methods, separation and detection techniques, and the availability of the new certified reference materials. The element speciation has more and more applications in various scientific areas. Both the elaboration of the measurement methods and usage of the research results should be interdisciplinary. The speciation investigations call for the mutual cooperation of chemical analysts with biologists and toxicologists [46].

Hyphenated methods provide new research opportunities [102, 103]. Their main advantages are extremely low detection and quantification limits, insignificant influence of the interferences in determinations, and very high determination precision and repeatability. Obviously, they also have limitations, such as the high price and complexity of the apparatus. Consequently, they are not normally available and used in the laboratories. Using hyphenated techniques requires full understanding of the analytical methodologies and apparatus operations. The systems are expensive and are used for scientific studies rather than routine analyses. Nonetheless, the development of these methods is becoming more and more important, which is corroborated by the growing number of applications and studies [104].

## Figures and Tables

**Table 1:** Application of LC-ICP-MS techniques in chromium, arsenic, antimony, and thallium speciation in water and sediment samples.

Analytes	Analytical column	Mobile phase	Method of separation and detection	Matrix	References
As^3+^, As^5+^, MMA, DMA	Hamilton PRP-X100 Dionex IonPac AS7, AG7	75 mM Na_3_PO_4_, 2.5–50 mM HNO_3_	HPLC-ICP-MS	Surface water, mining water, underground water	[54]

As^3+^, As^5+^, MMA, DMA, AB, AC	Waters IC-Pak CM/D Waters Guard-Pak CM/D	NaHCO_3_/Na_2_CO_3_, HNO_3_	HPLC-ICP-MS	Water	[56]

As^3+^, As^5+^, MMA, DMA, AB	Hamilton PRP-X100	20 mM NH_4_H_2_PO_4_	HPLC-ICP-DRC-MS	Polluted waters	[81]

As^3+^, As^5+^	Hamilton PRP X-100	Na_2_CO_3_	HPLC-ICP-MS	Surface water	[61]

As^3+^, As^5+^, MMA, DMA	Dionex IonPac AS7	HNO_3_	HPLC-ICP-MS	Waters	[82]

As^3+^, As^5+^	Wescan Anion-S C18	EDTA	HPLC-ICP-MS	River waters	[83]

As^3+^, As^5+^	Waters IC-Pak A HC	NaOH, KNO_3_	HPLC-ICP-MS	Water	[58]

As^3+^, As^5+^	Dionex IonPac AG12A/AS 12A	Disodium carbonate, sodium hydroxide, methanol	HPLC-ICP-MS	Iron rich water samples	[84]

As^3+^, As^5+^, MMA, DMA	Hamilton PRP X100	NH_4_NO_3_	HPLC-ICP-MS	Water	[62]

As^3+^, As^5+^, MMA, DMA, AB	Supelcosil LC-SCX	Pyridine, NaHCO_3_, Na_2_CO_3_	HPLC-ICP-MS	Aqueous extracts	[85]

As^3+^, As^5+^, MMA, DMA, AB	Dionex IonPac AS7	NH_4_H_2_PO_4_, NH_4_OH	HPLC-ICP-MS	Waters	[86]

As^3+^, As^5+^	Hamilton PRP X100	CH_3_COOH, NH_4_NO_3_, EDTA	HPLC-ICP-MS	Drinking water	[87]

As^3+^, As^5+^, MMA, DMA, AC, AB	Spherisorb ODS/NH_2_	5 mM NaH_2_PO_4_, 5 mM Na_2_HPO_4_,	HPLC-ICP-MS	Water	[57]

As^3+^, As^5+^, MMA, DMA	Hamilton PRP-X100	10–200 mM NH_4_H_2_PO_4_	HPLC-ICP-DRC-MS	Sediments	[77]

As^3+^, As^5+^, MMA, DMA, AB, AC	Develosil C30-UG-5, Chemcosorb 7SAX	Sodium butanesulfonate, malonic acid, tetramethylammonium hydroxide, metanol, ammonium tartrate	HPLC-ICP-MS	Environmental samples	[65]

As^3+^, As^5+^	Dionex IonPac AG-7 Hamilton PRP-X100	10 mM HNO_3_	HPLC-ICP-MS	Water	[33]

Sb^3+^, Sb^5+^,	Hamilton PRP-X100	20 mM EDTA, 2 mM KHP	HPLC-ICP-MS	Waters	[64]

Sb^3+^, Sb^5+^,	Hamilton PRP-X100	10 mM EDTA, 1 mM phthalic acid	HPLC-ICP-MS	Moat water	[68]

Sb^3+^, Sb^5+^,	Hamilton PRP-X100	20 mM EDTA, 2 mM KHP	HPLC-ICP-MS	Mineral water	[88]

Sb^3+^, Sb^5+^, TMSbCl_2_	Hamilton PRP-X100	20 mM EDTA, 2 mM KHP	HPLC-HG-AFS	Sea water,	[89]

Sb^3+^, Sb^5+^, TMSbO	Hamilton PRP-X100	2 mM phthalic acid 2 mM 4-hydroxybenzoic acid	HPLC-ICP-MS	Surface water	[69]

Sb^3+^, Sb^5+^, TMSbCl_2_	CETAC ION-120 Dionex IonPac AS14	2 mM NH_4_HCO_3_, 1 mM tartaric acid, 1.25 mM EDTA	HPLC-ICP-MS	Tap water	[67]

Sb^3+^, Sb^5+^, TMSbCl_2_, TMS(OH)_2_	Hamilton PRP-X100, Hamilton PRPX-200, Supelcosil LC-SCX, Hamilton PRP1, Phenomenex Intersil 5 ODS	KH_2_PO_4_/K_2_HPO_4_ 0.5–5 mM, KHCO_3_/K_2_CO_3_ 1–50 mM, Pyridine, 2,6 dicarboxylic acid (PDCA): 5–20 mM EDTA, 5–50 mM HNO_3_, 1–4 mM Ethylenediamine	HPLC-ICP-MS	Environmental samples	[90]

Sb^3+^, Sb^5+^	Synchropak Q300	5 mM EDTA, 2 mM phthalic acid	HPLC-ICP-MS	Tap water	[66]

Sb^3+^, Sb^5+^, TMSbCl_2_	Hamilton PRP-X100, Dionex IonPac AS4A-SC	12 mM tetra-methylammonium hydroxide 3 mM tetra-methylammonium hydroxide	HPLC-ICP-MS	Environmental samples	[91]

Sb^3+^, Sb^5+^	Dionex IonPac AS-7	1 mM Phtalic acid, 10 mM EDTANa_2_	IC-ICP-MS	Water and bottom sediments	[49]

Tl^1+^, Tl^3+^	Dionex IonPac CG12A	0.015 M HNO_3_	IC-ICP-MS	Water	[92]

Tl^1+^, Tl^3+^	Dionex IonPac AG12A CG12A	HNO_3_, HCl	IC-ICP-MS	Water	[70]

Tl^1+^, Tl^3+^	Dionex IonPac AS7	1.5 mM phtalic acid, 10 mM EDTA, 15 mM HNO_3_, 2 mM DTPA	IC-ICP-MS	Water, bottom sediments	[44]

Cr^3+^, Cr^6+^, Se^4+^, Se^6+^	Dionex IonPac AG-11 Dionex IonPac AS-11	20 Mm NaOH	IC-ICP-DRC-MS	Surface water, groundwater, tap waters	[32]

Cr^3+^, Cr^6+^	Waters Guard-Pak CM/D Waters IC-Pak A	0.4–40 mM HNO_3_	IC-ICP-DRC-MS	Sludge	[55]

Cr^3+^, Cr^6+^	Waters IC-Pak CM/D Waters Guard-Pak CM/D	0.4–40 mM HNO_3_	IC-ICP-DRC-MS	Water	[93]

Cr^3+^, Cr^6+^	G3145A/101 G3145A/102	30 mM NH_4_H_2_PO_4_	IC-ICP-MS	Sludge	[94]

Cr^3+^, Cr^6+^	G3145A/101 G3145A/102	20 mM NH_4_NO_3_	IC-ICP-DRC-MS	Saline water with a high content of Cl^−^	[95]

Cr^3+^, Cr^6+^	Shodex RS-pak NN-814 4DP	90 mM (NH_4_)_2_SO_4_ 10 mM NH_4_NO_3_	IC-ICP-DRC-MS	Water	[96]

Cr^3+^, Cr^6+^	Prepared in the laboratory	0.70 M HNO_3_	IC-ICP-MS	Seawater	[97]

Cr^3+^, Cr^6+^	Dionex IonPac CS5	2 mM PDCA + 2 mM NaHPO_4_ + 1 mM NaI + 5 mM CH_3_COONH_4_	IC-ICP-MS	Drinking water	[98]

Cr^3+^, Cr^6+^	Excelpak ICS-A23	1 mM EDTA-2NH_4_ + 10 mM H_2_C_2_O_4_	IC-ICP-MS	Drinking water, sludge	[99]

Cr^3+^, Cr^6+^	Dionex IonPac CS5	PDCA + (NH_4_)_2_HPO_4_ + CH_3_COONH_4_ + NH_4_OH + NH_4_I	IC-ICP-MS	Water	[100]

Cr^3+^, Cr^6+^	Dionex IonPac CS5A	40 mM MgSO_4_ + 30 mM HClO_4_	IC-ICP-MS	Drinking water, sludge	[101]

Cr^3+^, Cr^6+^	Dionex IonPac AG7	0.1 M NH_4_NO_3_ pH = 4 0.8 M HNO_3_	IC-ICP-MS	Water and bottom sediments	[49]

## Abbreviations

CE: Capillary electrophoresis
CNS: Central nervous system
DMA: Dimethyloarsenine
DMPS: 2,3-Dimercapto-1-propanesulfonic acid
DNA: Deoxyribonucleic acid
EDTA: Ethylenediaminetetraacetic acid
FDA: Food and Drug Administration
FIA-AAS: Flow injection analysis atomic absorption spectrometry
GC: Gas chromatography
HPLC: High performance liquid chromatography
HPLC-ICP-MS: High performance liquid chromatography inductively coupled plasma-mass spectrometry
HPLC-ICP-SF-MS: High performance liquid chromatography sector field inductively coupled plasma-mass spectrometry
IARC: International agency for research on cancer
ICP-CC-QMS: Inductively coupled plasma collision cell quadrupole mass spectrometry
ICP-DRC-QMS: Inductively coupled plasma dynamic reaction cell quadrupole mass spectrometry
ICP-MS: Inductively coupled plasma-mass spectrometry
ICP-SF-MS: Inductively coupled plasma sector field mass spectrometry
IPRP-ICP-MS: Ion-pairing reversed phase chromatography inductively coupled plasma mass spectrometry
LC: Liquid chromatography
LC-ICP-MS: Liquid chromatography inductively coupled plasma-mass spectrometry
LC-MC-ICP-MS: Liquid chromatography-multicollector inductively coupled plasma-mass Spectrometry
LOD: Limit of detection
MC-ICP-MS: Multicollector inductively coupled plasma-mass spectrometry
MMA: Monomethylarsonic acid
RNA: Ribonucleic acid
RSD: Relative standard deviation
TBAH: Tetrabutylammonium Hydroxide
TMSb: Trimethylantimony
TMSbO: Trimethylstiboxide
TSbCl_2_: Trimethlyantimony dichloride.
